# Short Nighttime Sleep Duration and High Number of Nighttime Awakenings Explain Increases in Gestational Weight Gain and Decreases in Physical Activity but Not Energy Intake among Pregnant Women with Overweight/Obesity

**DOI:** 10.3390/clockssleep2040036

**Published:** 2020-11-14

**Authors:** Abigail M. Pauley, Emily E. Hohman, Krista S. Leonard, Penghong Guo, Katherine M. McNitt, Daniel E. Rivera, Jennifer S. Savage, Danielle Symons Downs

**Affiliations:** 1 Exercise Psychology Laboratory, Department of Kinesiology, The Pennsylvania State University, 201 Old Main, University Park, PA 16802, USA; amp34@psu.edu (A.M.P.); kbl5167@psu.edu (K.S.L.); 2 Center for Childhood Obesity Research, The Pennsylvania State University, 129 Noll Laboratory, University Park, PA 16802, USA; eeh12@psu.edu; 3 School of Engineering of Matter, Transport, Energy, Arizona State University, Tempe, AZ 85287, USA; penghong.guo114@gmail.com (P.G.); daniel.rivera@asu.edu (D.E.R.); 4 Center for Childhood Obesity Research, Department of Nutritional Sciences, The Pennsylvania State University, 201 Old Main, University Park, PA 16802, USA; kmm6054@psu.edu (K.M.M.); jfs195@psu.edu (J.S.S.); 5 Department of OBGYN, Penn State College of Medicine, 700 HMC Crescent Road, Hershey, PA 17033, USA; 6 Kinesiology and Obstetrics and Gynecology, Department of Kinesiology, College of Health and Human Development, The Pennsylvania State University, University Park, PA 16801, USA

**Keywords:** pregnancy, sleep, energy balance, gestational weight gain, energy intake, physical activity, overweight, obesity

## Abstract

Pregnant women are at a high risk for experiencing sleep disturbances, excess energy intake, low physical activity, and excessive gestational weight gain (GWG). Scant research has examined how sleep behaviors influence energy intake, physical activity, and GWG over the course of pregnancy. This study conducted secondary analyses from the Healthy Mom Zone Study to examine between- and within-person effects of weekly sleep behaviors on energy intake, physical activity, and GWG in pregnant women with overweight/obesity (PW-OW/OB) participating in an adaptive intervention to manage GWG. The overall sample of *N* = 24 (*M* age = 30.6 years, *SD* = 3.2) had an average nighttime sleep duration of 7.2 h/night. In the total sample, there was a significant between-person effect of nighttime awakenings on physical activity; women with >1 weekly nighttime awakening expended 167.56 less physical activity kcals than women with <1 nighttime awakening. A significant within-person effect was also found for GWG such that for every increase in one weekly nighttime awakening there was a 0.76 pound increase in GWG. There was also a significant within-person effect for study group assignment; study group appeared to moderate the effect of nighttime awakenings on GWG such that for every one increase in weekly nighttime awakening, the control group gained 0.20 pounds more than the intervention group. There were no significant between- or within-person effects of sleep behaviors on energy intake. These findings illustrate an important need to consider the influence of sleep behaviors on prenatal physical activity and GWG in PW-OW/OB. Future studies may consider intervention strategies to reduce prenatal nighttime awakenings.

## 1. Introduction

**Figure 1:**
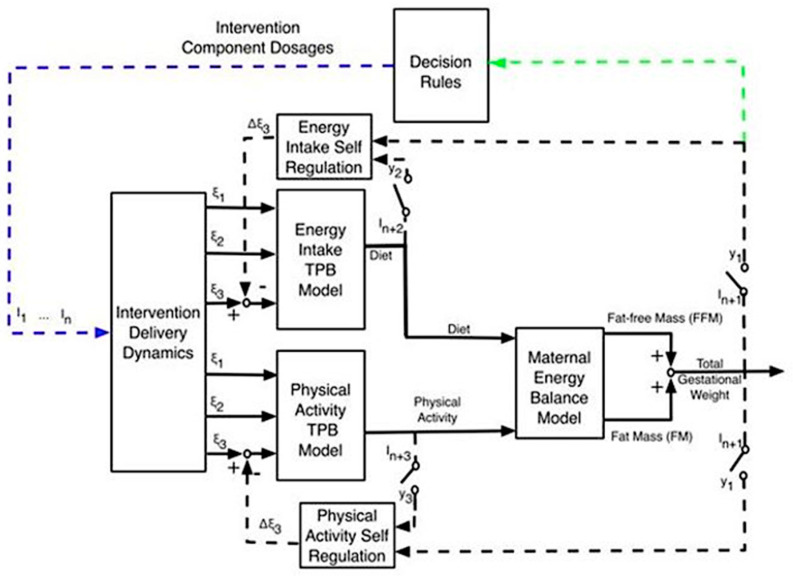
Dynamical model of energy balance. TPB: Theory of Planned Behavior; I_1_…I_n_: Intervention components; i: exogenous variables that serve as inputs for behavioral models; y_i_: system outputs; ξ_1_: Behavioral belief × evaluation of outcome; ξ_2_: Normative belief × motivation to comply; ξ_3_: Control belief × power of control belief; I_1_: Healthy Eating Education; I_2_: Healthy Eating Weekly Plan; I_3_: Healthy Eating Active Learning; I_4_: Goal Setting; I_5_: Physical Activity Education; I_6_: Physical Activity Weekly Plan; I_7_: Physical Activity Session; I_8_: Daily Weight Scale; I_9_: Dietary Record; I_10_: PA monitor output. This model includes the following: (1) a two-compartment energy balance model predicting changes in body mass as a result of energy intake and physical activity, (2) two Theory of Planned Behavior models describing how energy intake (diet) and physical activity are affected by behavioral variables, (3) a program delivery module relating magnitude and duration of components to inflows of the Theory of Planned Behavior models, and (4) two self-regulation units modeling how success expectancies in the intervention influence one’s goal achievement motivation. Within the Healthy Mom Zone intervention, the output from this model informs decision rules (green arrow) to provide the framework for making adaptations to the intervention component dosages for each individual participant (blue arrow).

Prenatal sleep behaviors (e.g., nighttime sleep duration and awakenings, daytime nap duration) fluctuate over the course of pregnancy [1,2,3]. Scant research, however, has examined prenatal sleep behaviors in relation to maternal outcomes such as gestational weight gain (GWG) [4]. Excessive GWG is a major public health concern among pregnant women, especially pregnant women with overweight/obesity (PW-OW/OB) because it can lead to adverse pregnancy outcomes for mother and infant (i.e., gestational diabetes, cesarean section, preterm birth, fetal growth restriction) [5,6,7,8,9,10,11,12,13]. Regulating GWG has largely focused on moderating energy intake and increasing physical activity behaviors. As such, Symons Downs and colleagues expanded on a pregnancy energy balance model to also include planned and self-regulatory behaviors to predict GWG (Figure 1) [5,6,7,8,9,10,11,12,13,14,15,16,17,18,19,20]. However, there is emerging interest in understanding the extent to which prenatal sleep behaviors relate to components of this energy balance model to explain GWG.

Managing energy intake and physical activity kilocalories (kcal) is required to ensure adequate weight gain while not exceeding GWG guidelines (i.e., OW: 0.5–0.7 pounds/week, OB: 0.4–0.6 pounds (lb)/week) [6]. With respect to prenatal energy intake, guidelines suggest trimester-specific kcals (i.e., no increase in kcals/day in first trimester, additional 340 kcals/day in second trimester, additional 452 kcals/day in third trimester) and general guidance about choosing high quality foods over the course of pregnancy [21,22,23]. Pregnant women are also encouraged to engage in 150 min/week of moderate intensity physical activity [24,25]. In theory, meeting energy intake and physical activity recommendations should effectively regulate GWG. However, over 80% of PW-OW/OB exceed the weekly guidelines and gain nearly twice as much total GWG over pregnancy compared to their normal weight counterparts [6,25,26]. Existing evidence from randomized control trial interventions shows moderating energy intake and increasing physical activity can effectively regulate GWG in pregnant women with normal weight, however, there is some but limited impact on regulating GWG among PW-OW/OB due to lack of adherence to energy intake and physical activity recommendations [11,26,27]. The model of energy balance to regulate GWG does not take into account the extent to which prenatal sleep behaviors influence energy intake and physical activity despite the growing evidence to suggest that sleep behaviors may be influential [4,27,28,29,30,31]. For example, Pauley and colleagues systematically reviewed associations between prenatal sleep behaviors (e.g., nighttime sleep duration and awakenings, sleep quality, daytime nap duration) and energy intake, physical activity, and GWG [28]. They found that short nighttime sleep duration and a high number of nighttime awakenings were associated with poor eating behaviors, and good sleep quality was associated with good eating behaviors [28,29,30]. The only significant association and effect size was between sleep behaviors and physical activity such that normal (i.e., 7–9 h/night) to long (i.e., ≥10 h/night) sleep duration was significantly associated with high physical activity (*p* < 0.001, effect size = 0.13) [28,31]. There is also evidence to suggest that short sleep duration is associated with both insufficient and excessive GWG [28,32,33]. These equivocal findings suggest that sleep behaviors appear to be related to GWG, but further research is needed to elucidate the direction of associations, especially among PW-OW/OB who may be at increased risk for poorer sleep behaviors and higher GWG.

One gap in the literature is the lack of studies measuring prenatal sleep with objective devices such as actigraphy [34]. Another limitation is the limited number of studies assessing prenatal sleep behaviors, energy intake, physical activity, and GWG at the weekly level. This is important because energy intake (kcal goals/recommendations) and physical activity (kcal goals/minutes of activity) goals are based on weekly values and can impact GWG change from week to week [35,36]. Given the small amount of GWG that is recommended each week for PW-OW/OB (i.e., OW: 0.5–0.7 or OB: 0.4–0.6 lb/week), a critical aspect of regulating GWG is identifying modifiable factors (such as sleep behaviors) that may impact GWG on a weekly basis [35,36]. This study provides a novel opportunity to examine actigraphy measured prenatal sleep behaviors within the conceptual framework of the energy balance model to predict GWG over the course of pregnancy [18,19,20,37,38].

The purpose of this study was to conduct secondary data analyses from the Healthy Mom Zone Study, a theory-based, behavioral intervention that adapted the intervention dosage and intensity over time in PW-OW/OB to regulate GWG [39,40]. The 29-week intervention was tested in a feasibility randomized control trial. This study aimed to: (1) describe means of sleep behaviors across the total sample and by study group assignment (i.e., intervention vs. control), and (2) examine between- and within-person effects of weekly sleep behaviors on weekly energy intake, physical activity, and GWG among all women and by study group assignment [18,19,20,37,38]. Based on existing literature, it was hypothesized that: (1) decreases in weekly nighttime sleep duration, increases in the number of weekly nighttime awakenings, and increases in weekly daytime nap duration would predict increases in weekly energy intake and GWG, and decreases in weekly physical activity [4,29,31].

## 2. Methods

### 2.1. Participants

PW-OW/OB (*N* = 31) were recruited to participate in the Healthy Mom Zone intervention. Women were eligible to participate if they were aged between 18–40 years and had: (1) overweight/obesity (BMI range 25–45 kg/m^2^; >40 kg/m^2^ with physician consultation), (2) singleton pregnancy >8 weeks gestation, (3) physician’s consent to participate, and (4) were English-speaking, residing in or near Central Pennsylvania. Exclusion criteria were: (1) multiple gestation, (2) diabetes at study entry, (3) not having overweight/obesity, (4) severe allergies or dietary restrictions, (5) contraindications to prenatal physical activity, and 6) not residing in area for duration of study [24]. Of the *N* = 31 women, *n* = 3 had miscarriages prior to starting the intervention and *n* = 1 woman withdrew, resulting in a total sample of *N* = 27. Of the *N* = 27 women, *n* = 3 women had limited sleep and physical activity data and were excluded from the current analyses. There were no differences in demographics or the remaining study variables (i.e., energy intake and GWG) when these women were included versus when they were excluded. Thus, the current secondary analyses are based on a total sample of *N* = 24 PW-OW/OB.

### 2.2. Procedures

The Healthy Mom Zone study was approved by the Pennsylvania State University Institutional Review Board (IRB Study# 00003752) [37,38]. Women were recruited using on-site clinic, community-based, and Web-based strategies [37,38]. Interested women completed a baseline screening to determine eligibility. Women meeting these criteria were asked to complete a 30-min baseline session at ~8 weeks gestation at the University’s Clinical Research Center. Study procedures were explained to the participants and written informed consent was obtained for each woman. Women were instructed on how to record their diets 3 days/week using MyFitnessPal (a mobile application) and counseling and energy intake was estimated using a back-calculation method based on weight, physical activity, and resting energy expenditure [37,38,41,42,43]. From ~8–36 weeks gestation, women wore a wrist-worn activity monitor to measure their daily sleep and physical activity behaviors and an Aria Fitbit Wi-Fi scale to measure daily weight from home. Sleep behaviors, physical activity behaviors, and weights were transmitted via Bluetooth to research investigators each day and a study team member downloaded the data from the respective websites and uploaded the data to a secure data storage website service (REDCap) [39]. The study protocol included two groups: intervention and usual-care control. Women randomized to the intervention group met with a registered dietician during weekly one-on-one sessions to discuss education and provide counseling on energy intake, physical activity, GWG, and other lifestyle behaviors such as sleep, mental health care, and water intake. Women also received individualized calorie and physical activity goals [37,38]. Every 3–4 weeks, each woman in the intervention group had her GWG evaluated against the Institute of Medicine (IOM) recommendations. If her GWG was above the recommendations (i.e., 15–25 lb for women with overweight; 11–20 lb for women with obesity) [6], the intervention was adapted by increasing the intensity of the dosage [37,38]. Adaptations included hands-on activities such as healthy eating demonstrations (e.g., meal preparation, cooking) and guided physical activity sessions with a fitness instructor as well as self-regulation feedback [37,38]. The usual-care control group completed the same measurement protocol as the intervention group but did not receive the intervention components (e.g., education, counseling, or dosage adaptations [37,38]). A more detailed explanation of the Healthy Mom Zone Intervention can be found elsewhere [37,38].

### 2.3. Measures

Demographics and Personal Characteristics. At baseline, women self-reported personal demographics (i.e., age, race/ethnicity, income, marital status, education, employment) and current gestational age. Pre-pregnancy BMI (kg/m^2^) was calculated from self-reported pre-pregnancy weight and height.

Sleep Behaviors. Nighttime Sleep Duration, Number of Awakenings, and Daytime Nap Duration. The Jawbone wrist-worn activity monitor (UP by Jawbone, San Francisco, CA, USA) was used to measure nighttime sleep duration, number of awakenings, and total daytime nap duration for each woman, day and night. Data were electronically transferred to the Jawbone website and smartphone app. The study team downloaded each woman’s Jawbone data. Nighttime sleep duration was converted into minutes and averaged over a 7-day period to achieve a weekly average of nighttime sleep duration. The number of awakenings was averaged over a 7-day period to achieve a weekly average number of awakenings nightly. Daytime nap duration was converted into minutes and averaged over a 7-day period to achieve a weekly average. Women wore the activity monitor each day over the entire intervention; there were roughly 27 weeks of data for each participant. The sleep behaviors were also averaged pre- and post-intervention. The Jawbone activity monitor was compared to polysomnography in non-pregnant women and the Jawbone was in good agreement with polysomnography but did overestimate total sleep time by 26.6 ± 35.3 min [40]. The Jawbone had high sensitivity (0.96) and low specificity (0.37) [40]. The Jawbone activity monitor was also compared to an Actiwatch Spectrum in a non-pregnant population and results suggested the Jawbone overestimated total sleep time but was not significantly different when measuring wake after sleep onset [44].

Energy Intake. Energy intake was estimated using a validated back-calculation method [39,40,44,45,46]:

(1) $$EI_{est}\left(k\right)=\;\frac{-W\left(k+2T\right)+8W\left(k+T\right)-8W\left(k-T\right)+W\left(k-2T\right)}{12TK_{1}}-\;\frac{K_{2}}{K_{1}}\left(PA\left(k\right)+REE\left(k\right)\right)$$

The variables are as follows: *k* = 1, 2, …, *N* corresponding to day 1-day *N*. *W* represents maternal weight in kg while *T* represents sampling time which in this case was *T* = 1 day. PA represents physical activity in kcals. *REE* represents resting physical activity. This method was used to address concerns about under-/over-reporting of energy intake when using self-report food records [45]. Energy intake was predicted for each day over the intervention. This study used weekly averages of energy intake which were taken over a 7-day period for each week in the intervention.

Physical Activity. The same wrist-worn activity monitor measuring sleep behaviors was used to measure physical activity kcal (UP by Jawbone, San Francisco, CA, USA) [46,47]. Women wore the activity monitor 24 h/day over the entire intervention period. Each woman had her own wrist monitor that connected to her phone via a mobile app in order to view the data and use it as a self-monitoring tool. Physical activity kcal, obtained from the Jawbone, was averaged over a 7-day period for each week in the intervention as well as averaged pre- and post-intervention and monthly.

GWG. Weight was assessed daily using the Aria Fitbit Wi-Fi scale (Fitbit, San Francisco, CA, USA) over the entire intervention period. Women weighed themselves each day as soon as they woke up. The scale transmitted weights automatically to secure participant online accounts; online data were accessed and stored in REDCap [39]. Weekly GWG was calculated as the averaged weight at pre-intervention subtracted from the averaged weight at post-intervention. The Aria Wi-Fi weight scale is a valid and reliable measure of weight [48].

### 2.4. Data Analyses

Analyses were performed using SPSS v25.0 (IBM, Armonk, NY, USA) and SAS v9.4 (SAS Institute, Cary, NC, USA). Descriptive statistics were used to examine study means, standard deviations, and frequencies by the overall sample and study group assignment. All data were downloaded from a secure survey database and device website (REDCap) [39]. Individual week differences between study group assignments were compared using repeated measures analysis of variance (RM-ANCOVA). Cohen’s *d* effect sizes for the associations between sleep behaviors–energy intake, sleep behaviors–physical activity, and sleep behaviors–GWG were calculated [49]. Magnitudes of effect sizes were classified as small = 0.2, moderate = 0.5, large = 0.8 [49].

Multi-level modeling (MLM) was used to examine the changes in energy intake, physical activity, and GWG as a function of time-invariant and time-varying nighttime sleep duration and nighttime awakenings over the course of the intervention (i.e., weekly from pre- to post-intervention) [50]. Fixed effects models were examined. When addressing MLM assumptions of linearity, normality, homoscedasticity, and independence of observations, none were violated. To reduce error and bias, each model included grand centered means and person-centered means to examine between- and within-person effects. Grand centered means were calculated by subtracting the population mean from each subject’s individual value for each variable and were used for between-person effects. Person-centered means were calculated by subtracting the subject’s overall mean from each individual value for each variable and were used for within-person effects.

Weekly averages of sleep behaviors, energy intake, physical activity, and GWG were used to reduce the risk of missing data. Outliers were removed prior to analyses if the value was 3 SD above or below the mean and if each woman’s value was 3 SD above or below their mean [51]. Analyses conducted with and without outliers produced the same results. The study was powered on the primary outcome of GWG. Julious effectively argues that a sample size of *n* = 12 per group is adequate to assess feasibility [52]. Since there was an expected 20% drop-out rate based on previous pilot work recruiting 30 participants (15 per group) provided 80% power to detect a standardized effect size for GWG of 1.2 using a two-sided test with significance level of *p* = 0.05 [52,53,54]. Because the Healthy Mom Zone intervention study was purposely not powered to detect effects in secondary outcomes of sleep behaviors, energy intake, and physical activity, these are reported as exploratory with *p* < 0.10 consistent recommendations for pilot and feasibility studies.

## 3. Results

Demographics. The sample (*N* = 24; *M* age = 30.6 years, *SD* = 3.2) was homogenous; most participants were Caucasian, married, completed college, worked full time, were nulliparous, and had a family income of $40,000 or higher per year (Table 1). The gestational age of participants at study start was in the 1st trimester (*M* Gestational week = 10.1, *SD* = 1.6, *range* = 7–13 weeks) and pre-pregnancy BMI of participants was in the obese range (*M* BMI = 31.8 kg/m^2^, *SD* = 7.3, *range* = 24.1–48.9; 58.3% OW, 41.7% OB).

Missing Data and Outliers. Out of the 29 weeks of the study, there were a total of 696 viable weekly data points. There was a total of 161, 160, and 649 (23.1%, 22.9%, and 93.6%) missing weekly data points and a total of 29, 9, and 0 outliers removed (4.2%, 1.3%, and 0%,) for nighttime sleep duration, nighttime awakenings, and daytime nap duration, respectively. There was a total of 75, 151, and 97 (10.8%, 21.7%, 13.9%) missing weekly data points and a total of 0, 4, and 20 outliers removed (0%, 0.57%, and 2.9%) for energy intake, physical activity, and GWG, respectively. Due to the lack of daytime nap duration data (i.e., there were only 47 viable daytime nap data points due to lack of naps/no data collected from the activity monitor) further analyses were unable to be conducted. Missing data were mean replaced with a 4-day moving average such that if there were 4 data points around the missing data point, those values were averaged, and that missing data point was replaced. Mean replacement allows for more robust and complete data set to understand weekly changes [55].

**Figure 2:**
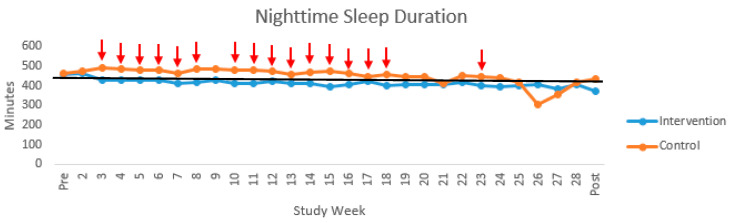
Nighttime sleep duration trajectory with marked study group assignment differences. Note. = significant/trending study group assignment differences; ____ = 7 h.

**Figure 3:**
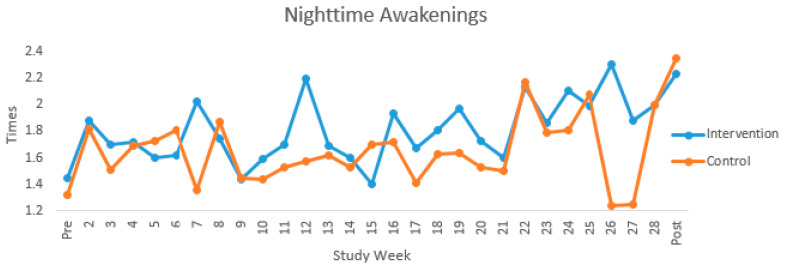
Nighttime awakenings trajectory.

Sleep Behavior Descriptives. In the overall sample, weekly nighttime sleep duration ranged throughout the study from 369.52 min/night (*SD* = 92.01) to 471.91 min/night (*SD* = 49.04). In comparison to nighttime sleep duration guidelines, weekly mean nighttime sleep duration hours were within the general recommended 7–9 h range up to Week 23. Although not significantly different from Weeks 1–23, nighttime sleep duration decreased under 7 h from Weeks 24 to Week 30 (post-intervention week) but none of the weekly average values were ≥8 h. Weekly nighttime sleep duration was significantly different between study assignment groups at Weeks 4, 5, 7, 8, 10, 11, 14–16 (*p* < 0.05) and trending at Weeks 3, 6, 12, 13, 18, and 23 (*p* < 0.1) with the control group having longer nighttime sleep duration; see Figure 2. There were no significant study group assignment differences in weekly nighttime awakenings; see Table 2 and Figure 3.

Multi-Level Modeling. There were no significant overall or weekly between-person, within-person, or study group effects of nighttime sleep duration or nighttime awakenings on energy intake (*p* > 0.10); see Table 3. There were no significant overall between-person, within-person, or study group effects of nighttime sleep duration on physical activity kcal (*p* > 0.10; effect sizes = 0.21, 0.08, 0.07). There was a significant weekly within-person effect such that when there was an increase in nighttime sleep duration, the overall sample expended 0.03 less physical activity kcals than the prior week (*p* = 0.03; effect sizes = 0.61). Study group assignment appeared to moderate the effect of nighttime sleep duration on physical activity kcal such that with an increase of 30 min of nighttime sleep duration, women in the control group expended 2.7 physical activity kcals more than the previous week compared to women in the intervention group (*p* < 0.001; effect size = 0.06). When comparing women with weekly nighttime awakenings >1 to women with weekly nighttime awakenings <1, women with awakenings >1 expended 167.56 less physical activity kcals (*p* = 0.06; effect size = 0.41) than women with <1 awakening. Within-person increases in nighttime awakenings were associated with increases in physical activity kcal such that when there was an increase in 1 nighttime awakening, the overall sample expended 2.44 less physical activity kcals than the prior week (*p* = 0.03; effect size = 0.45); see Table 4. There were no significant overall between-person or within-person effects of nighttime sleep duration on GWG (*p* > 0.10; effect sizes = 0.05, 0.10). However, Study group assignment appeared to moderate the effect of nighttime sleep duration on GWG such that when there was a 30 min increase in nighttime sleep duration, women in the control group gained 0.06 lb more than the previous week compared to women in the intervention group (*p* < 0.01, effect size = 0.74). A significant within-person effect was also found for GWG such that for every increase in 1 weekly nighttime awakening there was 0.76 lb increase in GWG (*p* = 0.07, effect size = 0.37). There was a significant weekly within-person effect such that an increase in 1 nighttime awakening, women gained 0.21 lb more than the previous week (*p* < 0.01, effect size = 1.42). Furthermore, study group assignment appeared to moderate the effect of nighttime awakening on GWG such that for every 1 increase in weekly nighttime awakening, the control group gained 0.20 lb more than the intervention group (*p* < 0.01, effect size = 0.76); see Table 5. Nighttime awakenings explained less than 5% of the variance in GWG (*p* < 0.05), which could be due to other factors (e.g., energy intake, physical activity, and potentially other sleep behaviors).

## 4. Discussion

The purposes of this study were to describe means of prenatal sleep behaviors and examine whether weekly sleep behaviors explained energy intake, physical activity, and GWG in a sample of PW-OW/OB randomized to an intervention study to regulate GWG. On average, the overall sample was within the 7–9 h sleep guidelines (7.23 h/night) and interestingly, women in the control group slept significantly longer than women in the intervention group. In the total sample, there was a significant between-person effect of nighttime awakenings on physical activity; women with >1 weekly nighttime awakening expended 167.56 less physical activity kcals than women with <1 nighttime awakening. A significant within-person effect was also found for GWG such that for every increase in one weekly nighttime awakening there was 0.76 lb increase in GWG. Study group assignment appeared to moderate the within-person effect of nighttime awakenings on GWG such that the control group gained 0.20 lb more than the intervention group. There were no significant between- or within-person effects of sleep behaviors on energy intake. These findings illustrate an important need to consider the influence of sleep behaviors on prenatal physical activity and GWG in PW-OW/OB. Future studies may consider intervention strategies to reduce prenatal nighttime awakenings.

While the overall sample averaged just slightly over 7 h of sleep/night from baseline through intervention Week 23, nighttime sleep duration decreased to under 7 h/night (i.e., average of 397.1 min/night or 6.6 h/night) from Week 24 (e.g., 30–35 weeks gestation) to Week 30 (post-intervention assessment). This decline in sleep duration in the later study weeks, although not significantly different from the earlier study weeks, appears to correspond with the physical changes that women experience in later gestation such as increased weight, muscle soreness, and discomfort. In contrast to our assumption, the control group slept 3–77 min significantly longer than the intervention group over the course of the study. With respect to nighttime awakenings, the overall sample had fewer than three nighttime awakenings/night but values steadily increased over the course of the intervention and were similar between the intervention and control groups (i.e., <2 nighttime awakenings/night). Although the control group slept longer than the intervention group, study group also appeared to moderate the effect of nighttime awakenings on GWG. For every increase in one nighttime awakening, the control group gained 0.20 lbs more than the intervention group. Taken together, these findings suggest that sleeping longer was not necessarily beneficial for GWG in the presence of nighttime awakenings. For example, the longer sleep duration may have been due to accommodating for the increased nighttime awakenings. Further research is needed to explore this assumption.

Additionally, in contrast to our hypothesis, there were no significant between- or within-person, weekly, or study group assignment effects of nighttime sleep duration and/or nighttime awakenings on energy intake. This may be due to a lack of power to detect an effect on the secondary outcome of energy intake. These findings are in contrast to findings by Pauley and colleagues suggesting short sleep duration and a high number of nighttime awakenings were associated with poor eating behaviors and good sleep quality was associated with good eating behaviors [28,29,30]. Moreover, there is further evidence that suggests poor sleep quality is related to higher food intake and lower-quality food choices in women [56]. Nevertheless, there is need for future research with appropriately powered studies to examine the effect of prenatal sleep behaviors on energy intake in PW-OW/OB.

In partial support of the hypothesis and past literature [28,31], increases in nighttime sleep duration and nighttime awakenings explained between-person and within-person decreases in weekly physical activity although the effect sizes were small. Most notably, compared to women with ≤1 nighttime awakening per week, women with >1 weekly nighttime awakening expended 167.56 less physical activity kcals for each one weekly nighttime awakening increase. These findings suggest that reductions in physical activity may be due to increased daytime fatigue and daytime napping to accommodate for the disturbed nocturnal sleep caused by high number of nighttime awakenings [57,58]. These findings illustrate a potential need for targeted education and goals that relate nighttime awakenings with physical activity in PW-OW/OB. That is, by creating goals to reduce the number of awakenings, it may help PW-OW/OB increase physical activity to effectively manage GWG within the context of a GWG management intervention. However, more research is warranted to examine if this approach can impact the role of sleep behaviors on physical activity in PW-OW/OB.

In partial support of the hypothesis, increases in nighttime awakenings explained within-person, weekly, and study group assignment increases in GWG, but not between-person effects. These associations are in line with previous literature that has found associations between sleep disruptions and excessive GWG in PW-OW/OB [4]. Most importantly, there was an effect in the overall sample and for study group assignment such that increases in nighttime awakenings explained increases in weekly GWG in the overall sample and in the control group compared to the intervention group. Alarmingly, the increase in GWG for the overall sample due to increases in nighttime awakenings was over the recommended weekly GWG (0.4–0.7 lb/week) for this population. Since this study occurred mainly over the second and third trimesters (e.g., 28 weeks), the accumulation of 0.76 lb/week can lead to a total of 22 lb, which in turn results in GWG above the IOM guidelines and elevates the risks for further negative health outcomes. Furthermore, the control group’s GWG increased on average by 0.20 lb from the previous week over the course of the study compared to the intervention group, which cumulatively could also lead to weight gain above overall GWG recommendations. These findings suggest that the intervention education on sleep hygiene could have been successful in improving sleep in the intervention women and may have had an effect on slowing down the weekly rate of GWG. It is important to use these current findings to guide further development of education for women on adopting strategies to reduce nighttime awakenings such as increasing physical activity time, limiting food and drink prior to bed time to reduce changes of needing to urinate and heartburn, and limiting daytime naps. By doing so, the rate of weekly GWG may be reduced.

This study had several strengths, one of which is the unique insight regarding relationships between sleep behaviors and energy intake, physical activity, and GWG in a randomized control trial setting to effectively manage GWG in PW-OW/OB. This study also included multiple sleep behaviors that may be negatively affected during pregnancy. Previous literature has only examined nighttime sleep duration but examining nighttime awakenings provides additional insight into prenatal sleep behaviors. Additionally, this study focused on PW-OW/OB who are at high risk for poor sleep behaviors. There has been a lack of sleep studies in this population and these findings will be able to provide the rationale for future studies to examine sleep behaviors in this population. This study used objective measures of sleep behaviors which allows for advancements in intervention methodology to use in free-living and interventional settings with pregnant women. Furthermore, objective measures of physical activity and GWG were measured, but energy intake was calculated using a validated back-calculation method based on measured physical activity and GWG [42]. This method is advantageous as it reduces the cost, risk of bias, and under-/over-reporting, and was the next best option to objectively measured energy intake. This study collected variables on the weekly level in order to correspond with the weekly IOM GWG recommendations. However, this study was not without limitations. First, generalizability of these findings may be limited due to the homogenous sample of mostly Caucasian, middle-class, OW/OB pregnant women. Second, daytime nap duration could not be examined due to insufficient data. Lastly, because this was a pilot feasibility study, it was not specifically powered to detect significant effects of the secondary outcomes (e.g., sleep behaviors, energy intake, physical activity). Further research is needed to examine the effects of prenatal sleep behaviors on these factors in a fully powered trial to confirm our study findings.

In conclusion, there are three key findings of this study. First, while PW-OW/OB averaged 7 h of sleep/night, there was also an observed decline in nighttime sleep duration below 7 h around 30–35 weeks gestation suggesting that the unique aspects of later gestation may influence nighttime sleep duration. Second, compared to women with <1 weekly nighttime awakening, women with >1 weekly nighttime awakening expend significantly less physical activity kcals. Lastly, for every increase in one nighttime awakening, GWG increased 0.76 lb in the overall sample and 0.20 lb weekly in the control group compared to the intervention group, which is above the IOM guidelines. As such, these findings illustrate an important need to consider the influence of sleep behaviors on prenatal physical activity and GWG in PW-OW/OB. Future studies may consider intervention strategies to reduce prenatal nighttime awakenings. Intervention education may include strategies on promoting healthy and adaptable sleep behaviors (e.g., promoting healthy foods, limiting snacking/drinking close to bed, increasing physical activity), how to improve sleep efficiency (i.e., time spent asleep in bed), sleep latency (i.e., amount of time it takes to fall asleep), and reducing the amount of time spent awake during the night. It is also important to educate women about waking up during the night in order to prepare for life with children and other life changes. By doing so, future researchers may be able to improve physical activity behaviors and reduce weekly GWG in PW-OW/OB. Future research is needed to replicate and confirm these findings to fully understand how sleep behaviors influence energy balance to effectively manage GWG among PW-OW/OB within an intervention context.

## Figures and Tables

**Table 1:** Participant Characteristics (*N* = 24).

	*Mean*	*SD*	*N* (%)
Age	30.6	3.2	
Gestational Week at Study Entry	10.1	1.6	
Pre-pregnancy BMI	31.8	3.2	
OW			14 (58.3)
OB			10 (41.7)
Race			
White			24 (100)
Employment			
Full-Time			21 (87.5)
Other			3 (12.5)
Education			
High School			1 (4.2)
College			11 (45.8)
Graduate/Professional			12 (50.0)
Family Income			
$10–20,000			1 (4.2)
$20–40,000			5 (20.8)
$40–100,000			10 (41.7)
>$100,000			8 (33.3)
Marital Status			
Married			22 (91.6)
Single			1 (4.2)
Divorced			1 (4.2)
Parity			
Nulliparous			18 (75)
Primiparous			6 (25)

Note. *SD* = standard deviation; BMI = body mass index.

**Table 2:** Weekly Means and Standard Deviations of Nighttime Sleep Duration and Nighttime Awakenings.

	Nighttime Sleep Duration (min)									Nighttime Awakenings								
	*Overall*			*Intervention*			*Control*			*Overall*			*Intervention*			*Control*		
*Study Week*	*N*	*M*	*SD*	*N*	*M*	*SD*	*N*	*M*	*SD*	*N*	*M*	*SD*	*N*	*M*	*SD*	*N*	*M*	*SD*
Pre	15	460.97	43.32	5	459.33	20.68	10	461.78	52.22	16	1.37	1.04	6	1.45	1.20	10	1.32	1.00
2	19	471.91	49.04	8	465.50	36.23	11	476.57	57.91	19	1.85	0.79	8	1.88	0.42	11	1.82	0.99
3	20	460.58	75.03	10	428.90	43.32	10	492.27	88.18	20	1.60	0.79	10	1.70	0.82	10	1.51	0.79
4	21	456.64	53.07	11	429.46	27.80	10	486.54	59.19	21	1.70	0.71	11	1.72	0.89	10	1.69	0.49
5	21	454.84	52.90	11	432.12	38.18	10	479.84	57.22	21	1.66	0.73	11	1.60	0.75	10	1.73	0.75
6	20	454.33	59.63	11	432.30	28.11	9	481.25	77.30	20	1.70	0.80	11	1.62	0.90	9	1.81	0.69
7	21	436.63	47.70	11	411.83	33.59	10	463.90	47.16	21	1.71	0.74	11	2.02	0.78	10	1.36	0.55
8	21	450.06	67.54	11	419.63	49.46	10	483.54	71.01	21	1.80	0.76	11	1.74	0.79	10	1.87	0.76
9	22	457.11	96.72	11	428.07	33.05	11	486.15	129.22	22	1.45	0.67	11	1.44	0.55	11	1.45	0.80
10	23	450.91	58.74	10	413.85	47.00	13	479.41	51.43	23	1.51	0.71	10	1.59	0.56	13	1.44	0.81
11	22	451.95	59.06	9	412.46	37.74	13	479.29	56.35	22	1.60	0.72	9	1.70	0.54	13	1.53	0.84
12	21	450.69	71.13	10	421.49	45.27	11	477.24	81.52	21	1.86	0.94	10	2.19	0.90	11	1.57	0.92
13	22	437.97	54.41	9	411.80	38.68	13	456.08	57.58	22	1.65	0.70	9	1.69	0.73	13	1.62	0.71
14	23	445.00	54.82	10	413.67	52.31	13	469.10	44.83	23	1.56	0.56	10	1.60	0.48	13	1.53	0.63
15	22	438.36	85.34	10	396.35	78.49	12	473.37	76.95	22	1.56	0.76	10	1.40	0.76	12	1.70	0.76
16	21	438.96	64.96	9	405.50	42.31	12	464.05	69.05	21	1.81	0.76	9	1.93	0.63	12	1.72	0.86
17	20	437.27	60.04	9	422.61	31.35	11	449.26	75.58	20	1.53	0.81	9	1.67	0.83	11	1.41	0.82
18	21	432.44	66.65	9	400.49	44.59	12	456.40	71.89	21	1.71	0.77	9	1.81	0.89	12	1.63	0.70
19	22	427.57	62.62	10	407.43	62.49	12	444.35	60.12	22	1.79	0.69	10	1.97	0.43	12	1.64	0.84
20	22	428.58	58.48	10	408.48	41.07	12	445.32	66.91	22	1.62	0.48	10	1.73	0.51	12	1.53	0.47
21	19	410.76	90.25	10	408.57	64.10	9	413.19	117.00	20	1.55	0.74	10	1.60	0.57	10	1.50	0.91
22	18	431.91	65.83	10	417.19	61.32	8	450.32	70.66	18	2.15	0.92	10	2.13	0.82	8	2.17	1.10
23	18	422.56	54.33	9	399.93	51.12	9	445.18	50.07	18	1.82	0.75	9	1.86	0.51	9	1.79	0.96
24	16	414.96	72.02	9	396.55	56.95	7	438.62	86.50	16	1.97	0.61	9	2.10	0.67	7	1.81	0.53
25	13	409.86	98.60	7	401.24	63.77	6	419.93	135.00	13	2.03	0.79	7	1.99	0.50	6	2.08	1.10
26	8	370.36	92.01	5	408.32	31.71	3	307.09	134.21	8	1.90	0.90	5	2.30	0.26	3	1.24	1.29
27	4	369.52	50.54	2	381.78	65.54	2	357.25	52.58	4	1.56	0.83	2	1.88	1.24	2	1.25	0.35
28	3	412.02	21.61	2	409.17	29.76	1	417.73	-	2	2.00	0.00	1	2.00	-	1	2.00	-
Post	17	405.65	72.10	8	375.44	53.57	9	432.50	78.51	17	2.29	0.80	8	2.23	0.71	9	2.35	0.92

Note. *M* = mean; *SD* = standard deviation; - = no data to report.

**Table 3:** Coefficients for the Multilevel Models of Nighttime Sleep Duration and Nighttime Awakenings Predicting Weekly Energy Intake.

	Parameters	Estimate	Standard Error	*p*-Value	Effect Sizes
Nighttime Sleep Duration	Intercept	4572.56	1260.33	<0.01	
	Within-Person NSD	−0.36	0.22	0.13	0.33
	Between-Person NSD	−3.87	2.85	0.19	0.28
	Week	13.57	2.25	<0.0001 *	
	Study Group	346.47	280.56	0.23	
	Week by Study Group	−2.74	4.41	0.53	
	Within-Person NSD by Week	0.01	0.02	0.61	0.10
	Within-Person NSD by Study Group	0.33	0.53	0.53	0.13
	Within-Person NSD by Week by Study Group	0.03	0.06	0.67	0.09
Nighttime Awakenings	Intercept	3417.36	489.71	<0.0001	
	Within-Person Awakenings	41.93	27.57	0.13	0.31
	Between-Person Awakenings	−318.52	277.90	0.26	0.23
	Week	13.30	2.26	<0.0001 *	
	Study Group	59.08	257.97	0.82	
	Week by Study Group	−3.14	4.43	0.48	
	Within-Person Awakenings by Week	−4.05	10.88	0.71	
	Within-Person Awakenings by Study Group	−63.46	99.03	0.52	
	Within-Person Awakenings by Week by Study Group	4.34	6.53	0.51	0.14

Note. NSD = nighttime sleep duration; Study Group Assignment: 0 = Intervention, 1 = Control. * *p* < 0.05.

**Table 4:** Coefficients for the Multilevel Models of Nighttime Sleep Duration and Nighttime Awakenings Predicting Weekly Physical Activity.

	Parameters	Estimate	Standard Error	*p*-Value	Effect Sizes
Nighttime Sleep Duration	Intercept	883.38	409.57	0.04	
	Within-Person NSD	−0.07	0.17	0.69	0.08
	Between-Person NSD	−0.97	0.93	0.31	0.21
	Week	0.73	0.77	0.35	
	Study Group	−78.94	92.66	0.40	
	Week by Study Group	−2.22	1.51	0.14	
	Within-Person NSD by Week	−0.03	0.01	0.03 *	0.61
	Within-Person NSD by Study Group	0.11	0.34	0.74	0.07
	Within-Person NSD by Week by Study Group	0.09	0.03	<0.001 *	0.70
Nighttime Awakenings	Intercept	741.49	148.07	<0.0001	
	Within-Person Awakenings	2.72	9.37	0.77	0.06
	Between-Person Awakenings	−167.56	84.01	0.06 #	0.41
	Week	1.12	0.75	0.13	
	Study Group	−128.85	73.53	0.09^#^	
	Week by Study Group	−1.75	1.47	0.24	
	Within-Person Awakenings by Week	−2.44	1.12	0.03 *	0.45
	Within-Person Awakenings by Study Group	5.72	18.75	0.76	0.06
	Within-Person Awakenings by Week by Study Group	0.62	2.2	0.78	0.06

Note. NSD = nighttime sleep duration; Study Group Assignment: 0 = Intervention, 1 = Control. * *p* < 0.05, # *p* < 0.1.

**Table 5:** Coefficients for the Multilevel Models of Nighttime Sleep Duration and Nighttime Awakenings Predicting Weekly GWG.

	Parameters	Estimate	Standard Error	*p-*Value	Effect Sizes
Nighttime Sleep Duration	Intercept	1.73	1.65	0.31	
	Within-Person NSD	−9.9 × 10^−5^	0.002	0.96	0.10
	Between-Person NSD	−0.001	0.004	0.71	0.05
	Week	0.14	0.02	<0.0001 *	
	Study Group	0.09	0.41	0.83	
	Week by Study Group	0.07	0.04	0.09	
	Within-Person NSD by Week	2.4 × 10^−4^	2.2 × 10^−4^	0.29	0.22
	Within-Person NSD by Study Group	0.002	0.005	0.61	0.08
	Within-Person NSD by Week by Study Group	0.002	0.001	0.005 *	0.74
Nighttime Awakenings	Intercept	1.80	0.66	0.01	
	Within-Person Awakenings	0.76	0.42	0.07 ^#^	0.37
	Between-Person Awakenings	−0.39	0.37	0.31	0.22
	Week	0.12	0.02	<0.0001 *	
	Study Group	−0.05	0.35	0.88	
	Week by Study Group	0.05	0.04	0.19	
	Within-Person Awakenings by Week	0.21	0.03	<0.0001 *	1.4
	Within-Person Awakenings by Study Group	−0.03	0.83	0.97	0.01
	Within-Person Awakenings by Week by Study Group	0.2042	0.06182	0.001 *	0.67

Note. NSD = nighttime sleep duration; Study Group Assignment: 0 = Intervention, 1 = Control. * *p* < 0.05, # *p* < 0.1.
